# Cases of Hypnotic Suggestion

**Published:** 1893-05

**Authors:** 


### TWO SINGULAR CASES OF HYPNOTIC SUGGESTION.

The Paris journals report a singularly interesting case treated by M. Luys, at the hospital, La Charité:

On the first of last February, Eugénie B——, a young lady of twenty years, entered the hospital to be treated for a nervous disorder. She was besides afflicted with an enormous purple-colored blotch which extended from the left ear to the collar bone, and covered completely one side of her neck and one-half of her left cheek. Dr. Luys and his assistant, Dr. Gérard Encausse, conceived the idea of employing hypnotic suggestion to remove this disfiguring blotch.

The young lady was accordingly put to sleep by the usual method, and then the suggestion was made to her not to have any longer this stain upon her face and neck. Three days after the first suggestion there appeared on the neck, in the middle of the blotch, a white spot nearly a centimetre square. In this place the skin had recovered its natural color. The suggestion was repeated each day, and on the 28th of February the white spot in the neck had increased considerably in size; moreover the skin of the ear had become entirely white. Day by day the blotch grew smaller; it seemed to melt away, attacked at once in its centre and on the edge, the skin gradually resumed a natural tint, and, what is the essential point in the experiment, it remains so.

Dr. Ernould has also performed some cures quite as curious. This is one of his most recent cases:

One of our friends has a young daughter who, for more than fifteen years, has been afflicted with total deafness, the result of an illness. The distress of a young lady suffering from this cruel infirmity can be easily understood. Every means to restore her hearing had been tried, but in vain. The best specialists of Brussels had been consulted, and they had pronounced it impossible to relieve her; she was to be deaf forever. When M. X—— heard the cures by hypnotism mentioned, he resolved to make a trial of this means, saying that if it did no good, it could do no harm. Dr. Ernould induced the hypnotic sleep, and suggested to the young girl that she ought to hear. On the second day a slight improvement was noticed ; she could hear the ticking of a watch placed against her ear ; the next day she distinctly perceived certain noises made in an adjoining room. Day by day the improvement continued, and to-day, after fifteen days of treatment, Mlle. X— has completely recovered her hearing.—*Banner of Light.*

